# Fuzi-Baijiangcao Herb Pair Alleviates DSS-induced Ulcerative Colitis in Mice *via* Inhibiting the p38 MAPK/NF-κB/HIF-1α Signaling Pathway

**DOI:** 10.2174/0113862073342236250103112654

**Published:** 2025-04-15

**Authors:** Chengxin Sun, Yinghao Jiang, Xiaoyan Wang

**Affiliations:** 1 The First Clinical Medical College, Shandong University of Traditional Chinese Medicine, Jinan, Shandong, China;; 2 College of Traditional Chinese Medicine, Shandong University of Traditional Chinese Medicine, Jinan, Shandong, China

**Keywords:** Fuzi-Baijiangcao herb pair, ulcerative colitis, p38 MAPK/NF-κB/HIF-1α signaling pathway, network pharmacology, bioinformatics, experimental validation

## Abstract

**Background:**

Ulcerative colitis is an inflammatory intestinal tract disease that is complex and difficult to heal, and prone to recurrence. The Fuzi-Baijiangcao (FB) herb pair is a commonly used drug combination for the clinical treatment of UC in China. However, its underlying mechanisms have not been elucidated. In this study, we explored the active ingredients of the FB herb pair and its potential mechanisms in the treatment of UC.

**Methods:**

Firstly, the active ingredients and potential targets of *Aconitum carmichaelii* Debeaux (Fuzi) and *Patrinia villosa* (Thunb.) Juss (Baijiangcao) were obtained through network pharmacology. We identified differentially expressed genes (DEGs) in UC by bioinformatics analysis. Then, the common targets of the FB herb pair and UC were obtained, and the protein-protein interaction (PPI) network was constructed. Gene Ontology (GO) and Kyoto Encyclopedia of Genes and Genomes (KEGG) enrichment analyses were performed using R language. The potential mechanism of the herb pair was validated through DSS induced UC mouse model.

**Results:**

Thirty-four active ingredients corresponding to 546 potential targets were screened in the FB herb pair. Bioinformatics analysis revealed 2125 DEGs in UC. The GO and KEGG enrichment analyses showed that the FB herb pair was able to exert therapeutic effects by modulating multiple inflammatory pathways, including MAPK, HIF-1, and TNF. The results of animal experiments demonstrated that the FB herb pair could significantly downregulate the gene and protein expression of key molecules in the p38 MAPK/NF-κB/HIF-1α signaling pathway, thereby reducing the expression of pro-inflammatory factors and cell adhesion molecules, significantly improving the symptoms and intestinal pathology, and repairing the damage of the intestinal mucosa in the UC mice.

**Conclusions:**

FB herb pair has Apparent therapeutic effects on UC, and its mechanisms may be related to the regulation of the p38 MAPK/NF-κB/HIF-1α signaling pathway to reduce the expression of inflammatory factors and repair intestinal mucosal damage.

## 1 INTRODUCTION

Ulcerative colitis (UC) is a chronic inflammation of the intestinal tract, manifesting as recurrent abdominal pain, diarrhea, and mucopurulent bloody stool [1]. As a global intestinal disease, the prevalence of UC is increasing year by year worldwide. The pathogenesis of UC is mainly related to immune dysfunction, genetic susceptibility, and impaired intestinal epithelial barrier, and it is difficult to cure with current therapeutic methods [2]. UC is a complex chronic disease that seriously affects the normal life of patients and brings physiological and psychological harm [3]. Therefore, exploring new effective drugs and treatments is a major challenge for the contemporary medical community.

Clinical studies have shown that Chinese herbal medicine can effectively alleviate the symptoms of UC, reduce recurrences, and have fewer side effects [4]. The combination application of Chinese herbal medicine can improve clinical efficacy and reduce adverse reactions [5]. *Aconitum carmichaelii* Debeaux (Fuzi) has the effects of tonifying fire, assisting yang, and dissipating cold. Modern pharmacological studies have found that it has anti-inflammatory and analgesic biological effects [6]. *Patrinia villosa* (Thunb.) Juss (Baijiangcao) has the effects of activating blood and resolving stasis, expelling pus, and relieving pain. Studies have confirmed that Baijiangcao has anti-inflammatory, anti-cancer, and anti-tumor effects [7-9]. The Fuzi-Baijiangcao (FB) herb pair originates from the classic Chinese herbal formula Yiyi Fuzi Baijiang powder in the Synopsis of the Golden Chamber of the Han Dynasty, which is commonly used in the treatment of UC [10]. However, the specific mechanism of FB herb pair has not been fully elucidated.

Network pharmacology, as an emerging discipline, reveals the relationship between diseases, targets, and drugs in greater depth and is widely used in the field of Chinese herbal medicine [11]. Bioinformatics is an interdisciplinary subject that combines biology and computer science and is of great significance for the extraction of biological information and the study of the molecular mechanisms of diseases [12]. Chinese herbal medicine has the characteristics of complex components, multiple targets, and multiple mechanisms of action. The combined application of network pharmacology and bioinformatics can help to gain a deeper understanding of the targets of Chinese herbal medicine intervention in diseases and reveal the mechanisms of Chinese herbal medicine treatment from multiple perspectives.

In this study, network pharmacology and bioinformatics methods were utilized to reveal the potential targets and molecular mechanisms of FB herb pair in the treatment of UC, which were further validated through animal experiments, providing references and bases for the combined application of Chinese herbal medicine for the treatment of UC.

## 2 MATERIALS AND METHODS

### 2.1 Drugs and Reagents

DSS (molecular weight: 36000-50000) is a product of MP Biomedicals (Aurora, Ohio, USA). Aconitum carmichaelii Debeaux was received from Huqiao Pharmaceutical Co., Ltd., China. Patrinia villosa (Thunb.) Juss was received from Shandong Baiweitang Pharmaceutical Co., Ltd., China. Antibodies against phospho-p38 MAPK (p-p38 MAPK) (4511), p38 MAPK (8690), HIF-1α (36169), phospho-NF-κB p65 (p-NF-κB p65) (3033), and NF-κB p65 (8242) were purchased from Cell Signaling Technology, Inc. (Boston, USA). Anti-VCAM-1 antibody (ab134047) and anti-ICAM-1 antibody (ab222736) were obtained from Abcam Company (Cambridge, UK), and β-actin was purchased from Wuhan Sanying Biology Technology Co., Ltd. (Wuhan, China). IP Lysate (R0010/R0020), Ex-taq Enzyme kits (PC1100), and BCA protein assay kit (PC0020) were obtained from Beijing Solarbio Science and Technology Co., Ltd., China. TRIzol reagent and SYBR Green qPCR Master Mix were purchased from Shanghai ExCell Biology Technology Co., Ltd., China. The reverse transcription kit (PrimeScript RTreagent kit with gDNA Eraser) was purchased from TaKaRa Biotechnology Co., Ltd. (Osaka, Japan). ELISA kits for TNF-α (CSB-E04741m) and IL-17 (CSB-E04608m) were obtained from Cusabio Technology, Inc. (Houston, USA).

### 2.2 Target Prediction of FB Herb Pair Active Ingredients and UC

The active ingredients of Fuzi and Baijiangcao were obtained from the traditional Chinese medicine system pharmacology database (TCMSP, https://tcmspw.com/tcmsp.php), and the screening conditions were the oral bioavailability (OB) ≥ 30% and drug likeness (DL) ≥ 0.18. TCMSP and Swiss target prediction (http://www.swisstargetprediction.ch) were used to predict the potential targets of the drug ingredients. The screened target names were converted into gene names using the Uniprot (https://www.uniprot.org) database, and the obtained results were imported into Cytoscape 3.9.0 to visualize the “drug-ingredient-target” network. GEO database for selection of UC differentially expressed genes (DEGs) and the R package “limma” (R 4.2.2), with |Log FC| > 0.5 and *P* value < 0.05 as the screening criteria, and then the R package “pheatmap” was used to draw volcano maps and heat maps. The drug targets were intersected with DEGs taken through the Venny 2.1 online website (https://bioinfogp.cnb.csic.es) to obtain the common targets of the UC and FB herb pair. The common targets were imported into the STRING (https://cn.string-db.org) platform to get the relationships of protein interactions, Cytoscape 3.9.0 software was used to draw protein-protein interaction (PPI) network, and the core targets were screened out according to the degree value. Subsequently, the common targets were ranked by R language, and the histogram of the top 30 targets was plotted. Finally, the common targets were analyzed by Gene Ontology (GO) and Kyoto Encyclopedia of Genes and Genomes (KEGG) enrichment using the “clusterProfiler” package in R language. The common targets and pathway networks were then constructed by Cytoscape 3.9.0.

### 2.3 Animals and Experimental Design

Eighteen male C57BL/6 mice (6 weeks old, 16-19 g) were provided by Beijing Vital River Laboratory Animal Technology Co., Ltd., China (SCXK (Jing) 2021-0006). After being fed on a standard diet for 7 days in a controlled environment (50% -70% relative humidity, 22-24°C, and 12 h light and dark cycles), the mice were randomly divided into three groups (n = 6): the Normal group, the DSS group, and the DSS+FB group. The mice of the Normal group drank purified water, while the mice of the DSS and DSS+FB groups freely drank 2.5% DSS solution for 7 days. After the UC mice models were established, the DSS+FB group and DSS group drank purified water. The mice in the DSS+FB group received 0.546 g/kg/day of FB solution by gavage daily for 7 days, while the mice of the DSS group and the Normal group mice were gavaged with normal saline 0.5 ml every day. After the last gavage, the mice were anesthetized with 1.5% isoflurane gas, the blood of mice was collected and then centrifuged at 4°C for 15 minutes (3000 rpm). The supernatant was collected and stored at -80°C for estimating inflammatory cytokines. After the colonic tissues were taken out, the lengths of the colons were measured, and the feces were removed with saline. Some tissues were preserved in 4% paraformaldehyde for histopathological examination, while the other tissues were stored at -80°C for further analysis. The animal study protocol was approved by the Ethics Committee of Shandong University of Traditional Chinese Medicine (approval number: SDUTCM20230404001).

### 2.4 Assessment of the Disease Activity Index (DAI)

The DAI is commonly used to evaluate the severity of UC. During the experimental process, the body weight, stool consistency, and hematochezia of the mice were recorded every day and each symptom was scored according to the previous method [13]. The percentage of weight loss was classified as 0, 1-5%, 6-10%, 11-15%, and more than 15%, recorded as 0, 1, 2, 3, and 4, respectively. The stool consistency was categorized as normal, wet feces, pasty feces, semi-liquid, and watery feces, recorded as 0, 1, 2, 3, and 4, respectively. The hematochezia was divided into normal, slight, moderate, severe, and gross bleeding, recorded as 0, 1, 2, 3, and 4, respectively. The total score of the above parameters was divided by 3 to obtain the DAI [14].

### 2.5 Histopathological Analysis

The fresh colon tissues were fixed with 4% paraformaldehyde for 48 h, dehydrated, and embedded in paraffin wax, and then the trimmed wax blocks were sectioned (thickness 4 μm). After the sections were dewaxed and washed, they were stained with hematoxylin and eosin (H&E), dehydrated, and sealed. Finally, the upright white light microscope (Nikon, Japan) was used to observe and photograph the sections.

### 2.6 UPLC-Q-Orbitrap MS/MS Analysis

UPLC-Q-Orbitrap MS/MS (UltiMate 3000 RS) chromatograph and Q Exactive high-resolution mass spectrometer were used for analysis. The chromatographic conditions were as follows.

The chromatographic analysis was performed using an AQ-C18 column (150 × 2.1 mm, 1.8 µm, Welch) with a flow rate of 0.3 mL/min and a column temperature maintained at 35°C. The aqueous phase comprised a 0.1% formic acid aqueous solution, and the organic phase was methanol. The automatic sampler injected a volume of 5.0 μL, with the autosampler temperature set at 10.0°C. Gradient elution was carried out as follows: 0–1 min, 2% B; 1–5 min, 20% B; 5–10 min, 50% B; 10–15 min, 80% B; 15–20 min, 95% B; 20–27 min, 95% B; 27–28 min, 2% B; and 28–30 min, 2% B. The mass spectrometry conditions included a full mass/dd-MS2 detection method with positive and negative ion switching scanning. An electrospray ionization (ESI) source was utilized with a spray voltage of 3.2 kV, a sheath gas flow of 40 Arb, an auxiliary gas flow of 15 Arb, and a capillary temperature of 300°C.

The scanning range was 100.0 - 1500.0 m/z, and the resolution was 70,000 (full mass) and 17,500 (dd-MS_2_).

### 2.7 Enzyme-linked Immunosorbent Assay (ELISA)

The Concentrations of IL-17 and TNF-α in the supernatant were determined by ELISA kits according to the manufacturer's instructions. Additionally, using a microplate reader, the OD values were recorded at 450 nm.

### 2.8 Western Blotting Assay

The colon tissues were ground with liquid nitrogen and lysate, and phenylmethylsulfonyl fluoride (PMSF) was added. Furthermore, total protein was extracted from colon tissues, and protein concentration was determined by the BCA method. The protein was separated by SDS-PAGE and transferred onto the PVDF membrane. The membranes were washed three times, blocked with 5% bovine serum albumin at 25°C for 3 h, and incubated with corresponding primary antibody at 4°C overnight: p38 MAPK, p-p38 MAPK, NF-κB p65, p-NF-κB p65, HIF-1α, VCAM-1, ICAM-1 (all 1:1000), and β-actin (1:5000). The membranes were washed by TBST for 5 times and incubated with secondary antibody at room temperature for 1h. Subsequently, the ECL reagent was added, and images were captured using a chemiluminescence imaging system. All experiments were performed in triplicate.

### 2.9 Quantitative Real-Time Polymerase Chain Reaction (RT-qPCR)

Total RNA was extracted from the colon tissues with TRIzol reagent following the manufacturer's instructions and the RNA concentration was determined using an ultra-micro spectrophotometer (ExCell MINIDROP). Reverse transcript mRNA to obtain cDNA according to the instructions of the reverse transcription kit. Quantitative PCR was carried out using the SYBR Green PCR Master Mix. The sequences of primers are listed in Table **1**. The relative expression levels of mRNA were calculated by the 2^-ΔΔCT^ method. All experiments were performed in triplicate.

### 2.10 Statistical Analysis

All experimental data were analyzed using SPSS 24.0 software. The results were shown as mean ± standard deviation. The data between groups were analyzed using one-way ANOVA, and paired comparisons were made using the LSD method. An unpaired T-test was also used to compare data differences between the two groups. *p* < 0.05 was considered statistically significant.

## 3 RESULTS

### 3.1 Targets Corresponding to Active Ingredients of FB Herb Pair and DEGs of UC

A total of 34 eligible active ingredients of FB herb pair were identified in the TCMSP database (Supplementary Table **S1**), of which 13 active ingredients were derived from Baijiangcao and 21 active ingredients were derived from Fuzi (Supplementary Table **S2**). These active ingredients corresponded to 546 potential targets, and the above results were imported into Cytoscape 3.9.0 to draw the “drug-ingredient-target” network diagram consisting of 99 nodes and 603 edges (Fig. **1A**). Subsequently, the GSE38713 dataset was analyzed using R language, and a total of 2125 DEGs of UC were obtained, of which 1127 were upregulated, and 998 were downregulated. Using R language, the heatmap (Fig. **1C**) and the volcano plot (Fig. **1B**) were drawn according to DEGs.

One hundred and seven co-targets were identified between FB herb pair and UC by Venn diagram (Fig. **2A**), and the PPI network was constructed of potential therapeutic targets (Fig. **2B** and Supplementary Table **S3**). The larger the node area, the darker the color, and the higher the degree, indicating that the gene is more important. The top 30 targets of co-targets with the highest correlation between drugs and UC were shown in Fig. **2D**. Based on the above analyses, it was found that the key targets of UC were IL-1β, JUN, MMP9, HIF-1α, VCAM-1, ICAM-1, and PPAR-γ. GO and KEGG enrichment analyses were performed using the R language. The GO enrichment results indicated that the FB herb pair mainly acted on the apical part of the cell and the external side of the plasma membrane and affected leukocyte migration, response to xenobiotic stimulus, positive regulation of cytokine production by affecting the activity of endopeptidase, serine-type peptidase, and serine hydrolase (Fig. **2C**). A total of 49 pathways were identified through KEGG enrichment analysis, among which the AGE-PAGE signaling pathway, TNF signaling pathway, HIF-1 signaling pathway, IL-17 signaling pathway, and MAPK signaling pathway were most closely related to core target genes (Fig. **2E**). Then we found that FB herb pair could regulate disease targets through multiple inflammatory pathways, thereby exerting effects on UC.

### 3.2 Effects of FB Herb Pair on Disease Symptoms and Colon Pathology of UC Mice

Body weights and DAI scores of the mice were measured daily. Mice drinking the DSS solution lost weight over time and gradually gained weight after the FB herb pair intervention. There was a statistically significant difference in weight between the DSS+FB and DSS groups since the 12^th^ day (Fig. **3A** and Supplementary Table **S4**). The DAI scores of mice drinking DSS solution gradually increased, reaching a maximum on the 9^th^ day, and gradually decreased after the FB herb pair intervention (Fig. **3B** and Supplementary Table **S5**). Next, we observed the colon of mice and measured its length, and found that the length of the colon was significantly shorter in the DSS group compared to the Normal group (Fig. **3C**). Histological analysis showed that the colonic tissues of mice in the DSS group had extensive ulceration, a large number of necrotic mucosal epithelial cells, numerous necrotic intestinal glands in the lamina propria, hyperplasia of connective tissues, and accompanied by a large amount of inflammatory cells infiltration. Edema could be seen in the submucosal layer with sparsely arranged connective tissues. However, in the DSS+FB group, the colonic epithelial mucosa was partially repaired, the arrangement of glands was relatively regular, and the aggregation of inflammatory cells was reduced (Fig. **3D**). The above results indicated that FB herb pair played an important role in disease treatment and the recovery of colonic mucosal injury in UC mice.

### 3.3 Chemical Constituents of FB-Containing Serum

The chemical constituents of FB-containing serum were analyzed by the UPLC-Q-Orbitrap HRMS method. The results are shown in Fig. (**4**) and Supplemental Table S6. Based on the literature, information on fragmentation, and excimer peaks, a total of seven chemical constituents have been identified, including DL-Homoserine, D-(+)-Malic acid, Epinephrine, Deoxycholic acid, Pinolenic acid, Monoolein, and Stearic acid.

### 3.4 FB Herb Pair Inhibited p38 MAPK/NF-κB/HIF-1α pathway

The activation of the p38 MAPK/NF-κB/HIF-1α signaling pathway is closely related to the pathogenesis of UC. We analyzed the expression of key genes and proteins in the p38 MAPK/NF-κB/HIF-1α signaling pathway using western blotting and RT-qPCR. As shown in Fig. (**5**), the protein expression levels of p-p38 MAPK and p-NF-κB p65 in the colons of DSS-induced UC mice were significantly increased when compared to the Normal group. The expression of p-p38 MAPK and p-NF-κB p65 was significantly reduced in the DSS+FB group after drug treatment. However, there were no statistically significant differences in the mRNA and protein expression levels of p38 MAPK and NF-κB p65 among all groups. We found that the expression levels of HIF-1α, VCAM-1, and ICAM-1 in the colons of UC mice were significantly higher than those in the Normal group. After FB herb pair treatment, the expression levels of HIF-1α, VCAM-1, and ICAM-1 were significantly decreased when compared to the DSS group. These results suggested that the FB herb pair might inhibit the activation of the p38 MAPK/NF-κB/HIF-1α signaling pathway to treat UC.

### 3.5 FB Herb Pair Reduced the Levels of Inflammatory Cytokines

The levels of inflammatory cytokines in the supernatant were detected by ELISA. The expression levels of cytokines such as TNF-α and IL-17 significantly increased in DSS-induced UC mice compared with the Normal group. However, the expression levels of TNF-α and IL-17 significantly decreased after FB herb pair treatment (Fig. **6**). These showed that FB herb pair could modulate the overexpression of inflammatory cytokines in UC mice.

## 4 DISCUSSION

UC is a chronic inflammatory disease, and its pathogenesis has not been fully clarified. There are numerous adverse effects of Western medicine for UC [15, 16], while Chinese herbal medicine has fewer side effects and is safe and effective [17]. FB herb pair, which is commonly used in the clinical treatment of UC, was selected for this study. Firstly, we identified the targets of UC and drugs through network pharmacology and bioinformatics. Then, 107 common targets between UC and FB herb pair were obtained by analysis, the PPI network was constructed, and GO and KEGG enrichment analyses were performed. These results showed that FB herb pair could treat UC by modulating multiple inflammatory pathways, including p38 MAPK, NF-κB, and HIF-1α. Subsequently, we performed in vivo experiments for validation.

In this study, we found that FB herb pair could effectively alleviate diarrhea and blood in stool, reduce DAI scores, and improve intestinal pathology in UC mice induced by DSS. Then, the chemical constituents of FB-containing serum were analyzed by UPLC-Q-Orbitrap HRMS. DL-Homoserine, Pinolenic acid, and Monoolein could inhibit the NF-κB signaling pathway, reduce the expression of pro-inflammatory factors such as TNF-α and IL-1β, and exert anti-inflammatory effects [18-21]. Deoxycholic acid could activate intestinal cells, promote intestinal epithelial regeneration, and repair DSS-induced colonic mucosal damage in UC mice [22, 23].

The p38 MAPK signaling pathway can inhibit inflammatory cytokines, regulate macrophage apoptosis, and have effects such as limiting inflammatory response and immune regulation, which is closely related to the development of UC [24, 25]. As an important member of the MAPK family, p38 MAPK plays an important regulatory role in cellular inflammation, proliferation, apoptosis, and tissue repair [26]. P-p38 MAPK is a key member of the p38 MAPK signaling pathway, which reflects the activation status of the pathway [27]. It was found that the expression of p-p38 MAPK in patients with inflammatory bowel disease significantly increased compared to healthy individuals [28]. NF-κB p65 is a key downstream signal of the p38 MAPK signaling pathway, and the activity of NF-κB p65 depends on the activation of p38 MAPK [29]. P-p38 MAPK could activate NF-κB to increase the production and release of pro-inflammatory factors and exacerbate the inflammatory response [30]. Previous studies showed the expression levels of NF-κB p65 and p-NF-κB p65 significantly increased in UC rats compared to normal rats while inhibiting the p38 MAPK/NF-κB pathway could reduce the levels of inflammatory factors and repair colonic damage in UC [31, 32]. As a key transcription factor, HIF-1α plays an important role in regulating inflammatory responses. When inflammation occurred, the expression of HIF-1α elevated in inflammation-related tissues or cells [33, 34]. NF-κB p65 could bind to the promoter of HIF-1α and upregulate the expression of HIF-1α [35], while HIF-1α was able to induce the up-regulation of IL-23 receptor, thus affecting the expression of inflammatory factor IL-17 [36]. In addition, multiple studies showed that the expression of HIF-1α increased with the activation of NF-κB p65, and inactivation of NF-κB p65 could also downregulate the expression of HIF-1α [37, 38]. Consistent with the above studies, we found high expression levels of p-p38 MAPK, p-NF-κB p65, and HIF-1α in the colonic tissues of UC mice, and all of these were significantly reduced by the administration of FB herb pair. It was found that benzoylaconine, a constituent of Fuzi, could exert anti-inflammatory effects by inhibiting the expression levels of p-p38 MAPK, NF-κB p65, and HIF-1α in the MAPK and NF-κB signaling pathways, and suppressing the expression of pro-inflammatory factors [39, 40].

Cytokines have the functions of participating in inflammation response and regulating immunity, Playing an important role in UC disease progression [41, 42]. IL-17 and TNF-α are thought to be critical in causing impaired intestinal immunity and inducing inflammation [43, 44]. IL-17 is the key inflammatory mediator in numerous diseases such as infection, autoimmunity, and cancer [45]. IL-17 is mainly produced by activated T cells, which can promote T cell activation and stimulate epithelial cells, endothelial cells, and fibroblasts to produce various cytokines such as IL-6, granulocyte-macrophage stimulating factor (GM-CSF), and cell adhesion molecule 1 (CAM-1), and maintain the release of inflammatory mediators TNF-a and IL-6, thereby amplifying the inflammatory response [46]. TNF-α is able to destroy the immune system and trigger inflammation [47]. Excessive TNF-α in the intestine could cause damage to epithelial cells, exacerbate inflammation, and has a significant impact on the development of UC [48]. Studies have shown that the expressions of IL-17 and TNF-α increased significantly with the aggravation of the inflammatory response in the colonic mucosa [49, 50]. In addition, IL-17 could enhance the expression of granulocyte-colony stimulating factor and granulocyte/macrophage induced by the TNF-α in human colonic [51]. Previous studies found that the expression levels of IL-17 and TNF-α were significantly elevated in the colonic tissues of UC animals [52, 53], and the same results were obtained in our study. We also found that treatment with FB herb pair reversed the UC-induced high expression of IL-17 and TNF-α. Modern pharmacology suggested that luteolin and apigenin derived from Baijiangcao could effectively inhibit the expression of inflammatory factors such as IL-1β and IL-17 in UC mice [54, 55]. Aconicleistanthane A, the extract of Fuzi, inhibited the secretion of pro-inflammatory factors and protein expression of iNOS and had anti-inflammatory effects [56]. Alkaloids of A. carmichaelii, the main ingredients of Fuzi, could inhibit the secretion of inflammatory cytokines TNF-α, IL-1β, and IL-17A through the MAPK/NF-κB signaling pathway, and reduce the phosphorylation of p38 MAPK and JNK as well as the expression of NF-κB in the colonic tissues of mice, which played an important role in the treatment of UC [57, 58].

Intestinal mucosal injury is the major pathological change in UC, and the cell adhesion molecules ICAM-1 and VCAM-1 play an important role in inducing intestinal mucosal injury [59]. ICAM-1 is a glycoprotein and adhesion receptor that is low-expressed in normal cells and tissues, but its expression is rapidly upregulated in inflammatory responses [60-62]. ICAM-1 was able to mediate neutrophil infiltration into colonic tissues, leading to mucosal injury [63]. IL-17 could upregulate the expression of ICAM-1 in intestinal endothelial cells by stimulating epithelial cells and myofibroblasts [64]. Studies showed that the expression of ICAM-1 was significantly elevated in the serum of UC patients [65, 66]. VCAM-1 is mainly distributed on the surface of endothelial cells, epithelial cells, and macrophages, and it can induce colonic inflammatory infiltration by enhancing the adhesion of lymphocytes and macrophages to endothelial cells. Increased expression of VCAM-1 was crucial for the transition of UC from quiescent to active phase [67, 68]. It has been found that during inflammation process, as the expression levels of inflammatory factors TNF-α and IL-17 were upregulated, the expression of VCAM-1 also increased [69]. However, decreasing the expression levels of ICAM-1 and VCAM-1 could reduce intestinal mucosal injury [70]. In line with above findings, we found that the expression levels of ICAM-1 and VCAM-1 were significantly elevated in the intestinal tissues of UC mice. After treatment with FB herb pair, the expression levels of ICAM-1 and VCAM-1 in UC mice were significantly reduced, and the intestinal tissue injury was also repaired to a certain extent. Apigenin, the component of Baijiangcao, could inhibit the expression of cell adhesion molecules ICAM-1 and VCAM-1, and had therapeutic effects on UC [71].

## CONCLUSION

Moreover, our study demonstrates that FB herb pair has good therapeutic effects on UC, the mechanism may exert therapeutic effects by inhibiting the p38 MAPK/NF-κB/HIF-1α inflammatory signaling pathway to reduce the expression of inflammatory factors and repair intestinal tissue damage. These findings provide new insights into the therapeutic effects and potential mechanisms of FB herb pair on UC.

This study does have some limitations. We have not yet studied whether the FB herb pair affects the p38 MAPK/NF-κB/HIF-1α signaling pathway through some regulatory molecules (such as non-coding RNA), which is also the research content we will explore in the future. In addition, we only carried out animal experimental verification, and we will also verify it through cell experiments in future research. Furthermore, we will set up different herbal dose groups in the experiments in order to explore the relationship between the therapeutic effects of herbs and drug doses. These studies may contribute to an in-depth exploration of the mechanisms of FB herb pair for the treatment of UC.

## AUTHOR'S CONTRIBUTIONS

It is hereby acknowledged that all authors have accepted responsibility for the manuscript's content and consented to its submission. They have meticulously reviewed all results and unanimously approved the final version of the manuscript.

## Figures and Tables

**Fig. (1):**
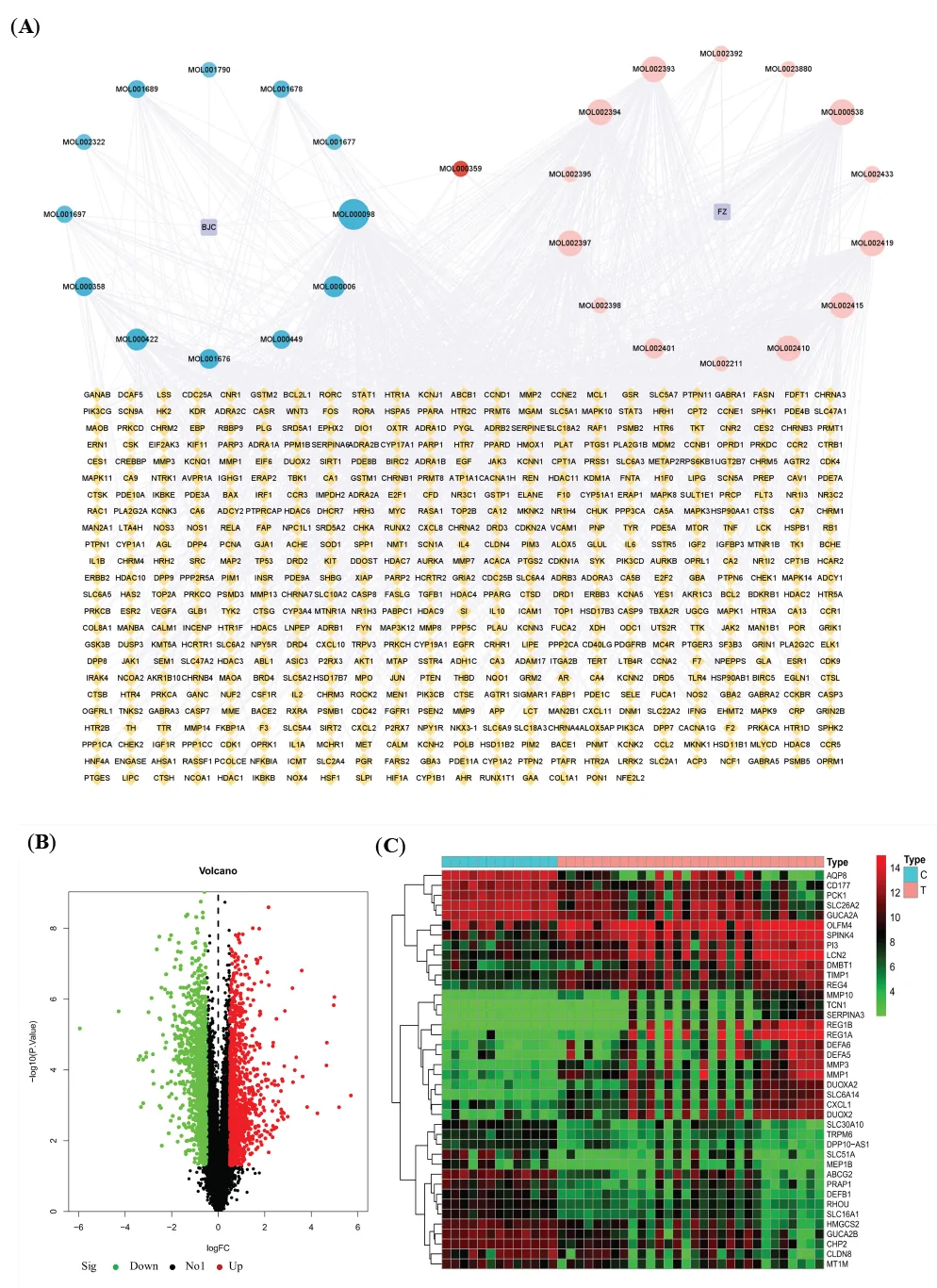
The active ingredient-target network and DEGs related to UC. (**A**) The ingredient-target network of FB herb pair. The blue nodes were the active ingredients of Baijiangcao, the pink nodes were the active ingredients of Fuzi, and MOL000359 was the common ingredient of Baijiangcao and Fuzi. The active ingredients of drugs were ranked based on degree values. The size of a node was proportional to the degree value. The larger the node, the stronger the interaction force between the drug ingredient and the target. **(B)** Volcano plot. The green dots indicated downregulated genes and the red dots indicated upregulated genes. (**C**) Heatmap. Red color showed upregulated expression and green color showed downregulated expression.

**Fig. (2):**
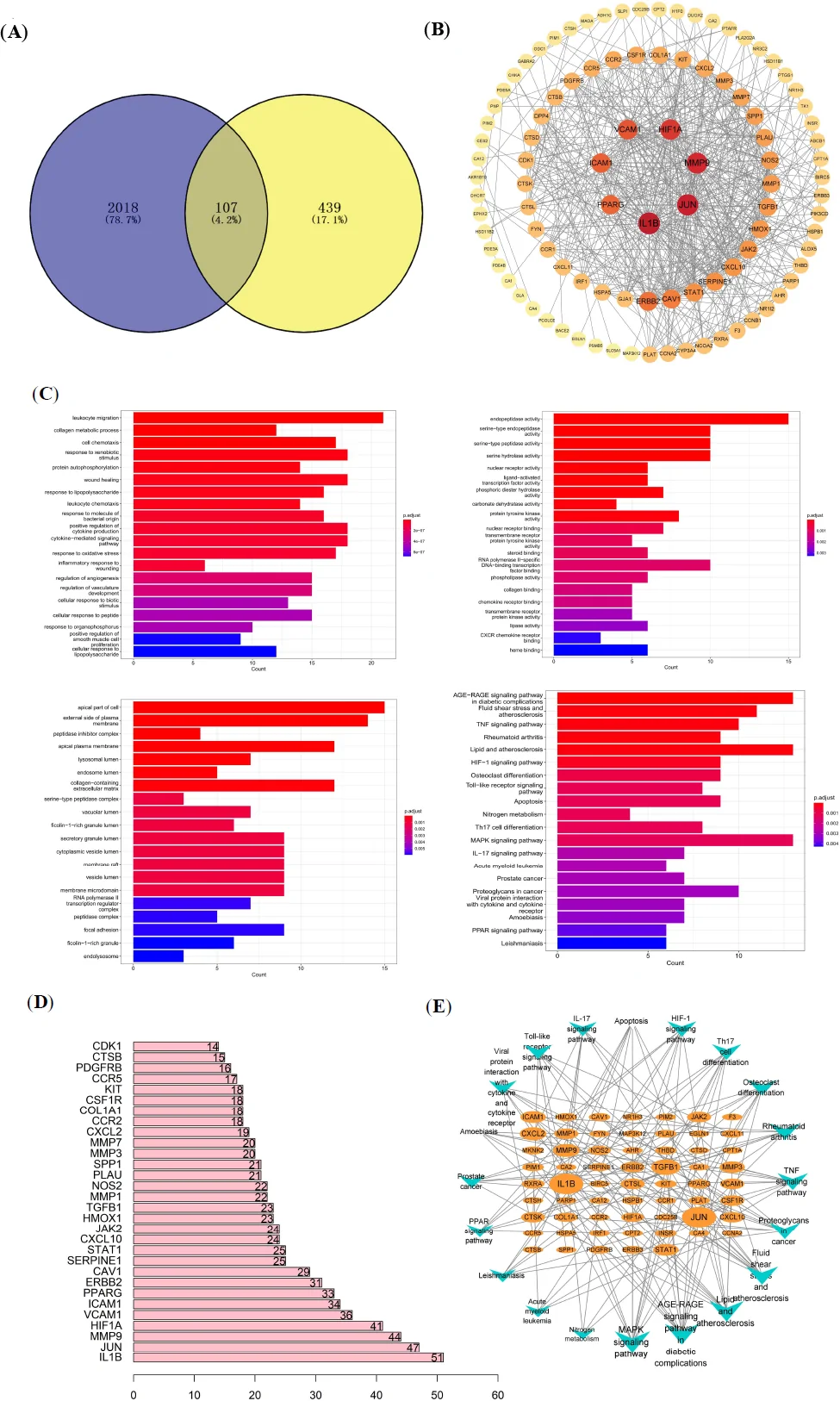
Analysis of common targets for FB herb pair and UC. **(A)** The Venn diagram of the intersecting targets between FB herb pair and UC**.** (**B**) PPI network of co-targets of FB herb pair and UC. **(C)** GO and KEGG pathway enrichment analyses. The results were ranked from high to low based on their importance**. (D)** The top 30 co-targets of FB herb pair in treatment of UC. (**E**) Target-pathway network**.** Signaling pathways were marked with blue nodes, and the co-targets of FB herb pair in the treatment of UC were shown as orange oval nodes.

**Fig. (3):**
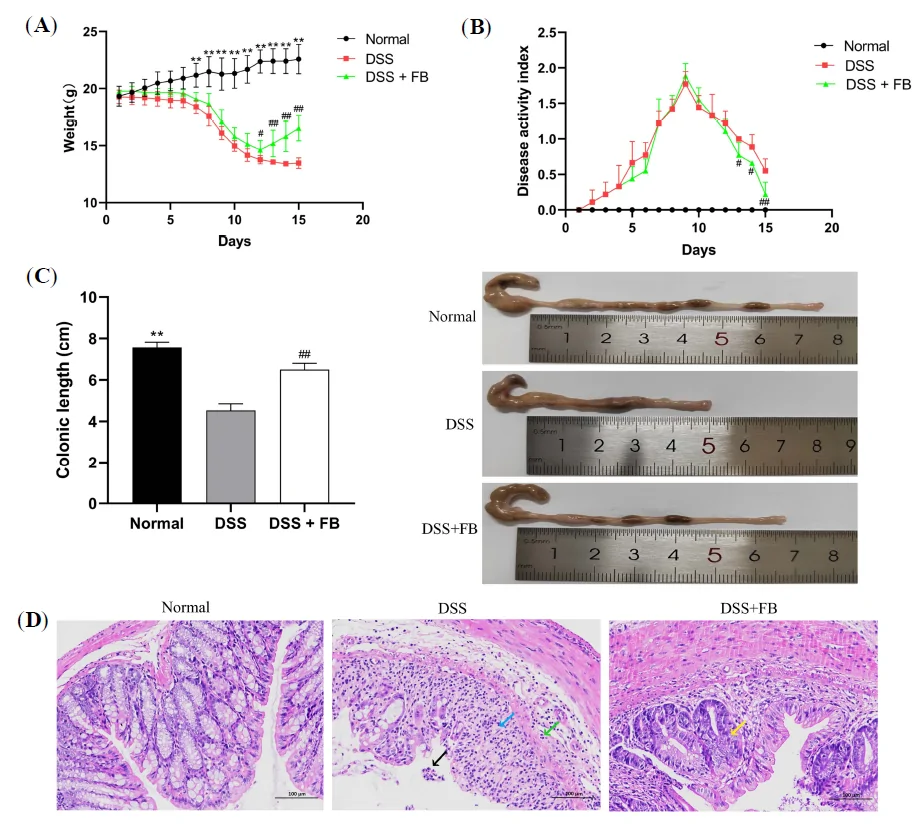
Effects of FB herb pair on disease symptoms and colon pathology of UC mice. (**A, B**) Body weights and DAI scores of mice in the experiment (n = 6). (**C**) Colon lengths of mice. (**D**) The histopathological alterations of colon tissues. Scale bar 100 μm. The black arrow indicated mucosal epithelial cell necrosis and shedding; the blue arrow indicated intestinal gland necrosis with connective tissue hyperplasia; the green arrow indicated massive granulocytic infiltration, and the yellow arrow indicated intestinal gland abnormalities. All values were expressed as mean ± SD. ^**^p < 0.01, the Normal *vs.* DSS group; ^#^*p* < 0.05 and ^##^*p* < 0.01, the DSS+FB *vs.* DSS group.

**Fig. (4):**
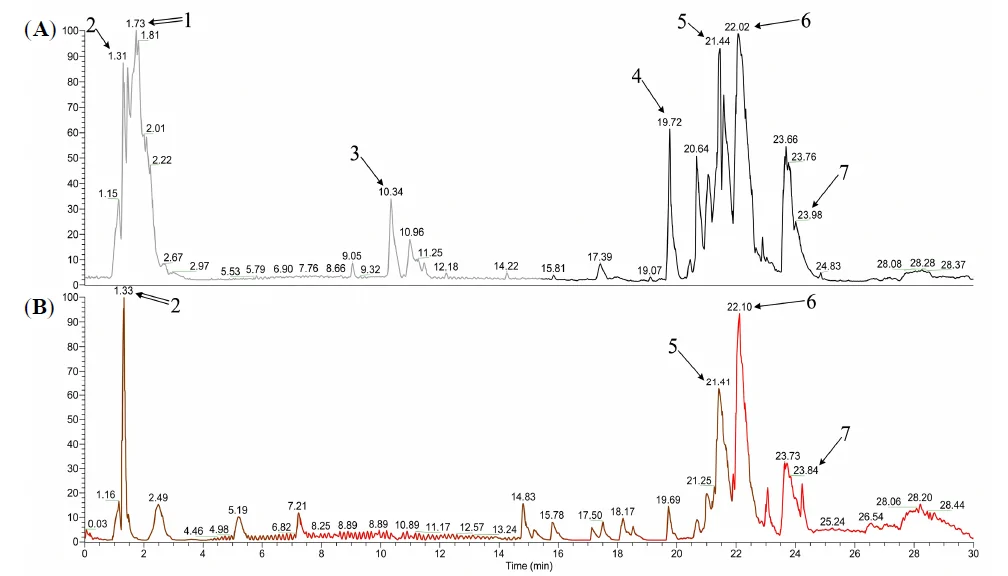
Positive (**A**) and negative (**B**) ion diagram of FB-containing serum. Peak **1**: DL-Homoserine, **2**: D-(+)-Malic acid, **3**: Epinephrine, **4**: Deoxycholic acid, **5**: Pinolenic acid, **6**: Monoolein, **7**: Stearic acid.

**Fig. (5):**
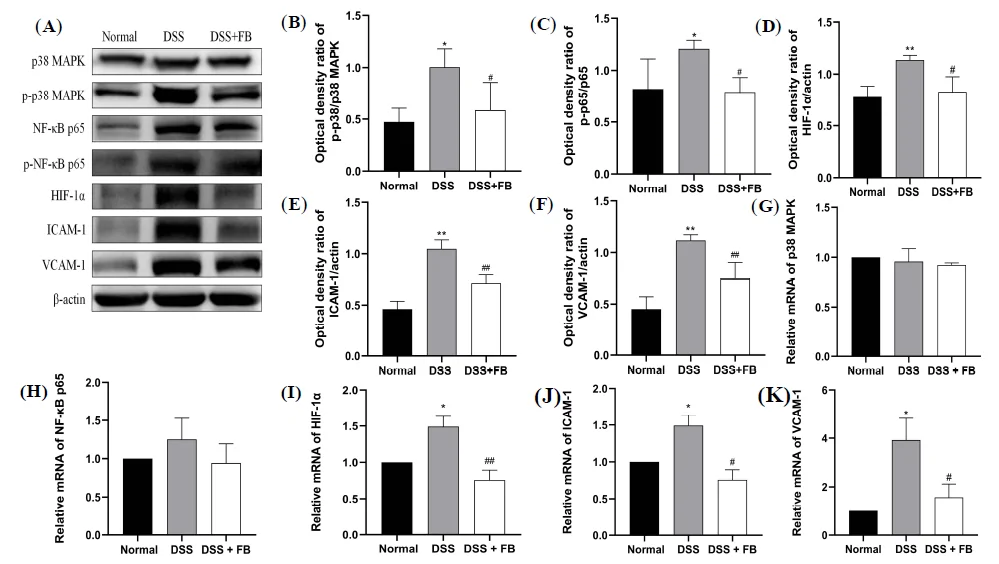
Effects of FB herb pair on p38 MAPK/NF-κB/HIF-1α signaling pathway. **A-F** The protein expression levels of p38 MAPK, p-p38 MAPK, NF-κB p65, p-NF-κB p65, HIF-1α, ICAM-1, and VCAM-1 in colon tissues of UC mice were detected by Western blot. RT-qPCR was used to detect the expression of p38 MAPK (**G**), NF-κB p65 (**H**), HIF-1α (**I**), ICAM-1 (**J**), and VCAM-1 (**K**). All values were expressed as mean ± SD, n = 3. ^#^*p* < 0.05 and ^##^*p* < 0.01 *vs.* the DSS group; ^*^p < 0.05 and ^**^p < 0.01 *vs.* the Normal group.

**Fig. (6):**
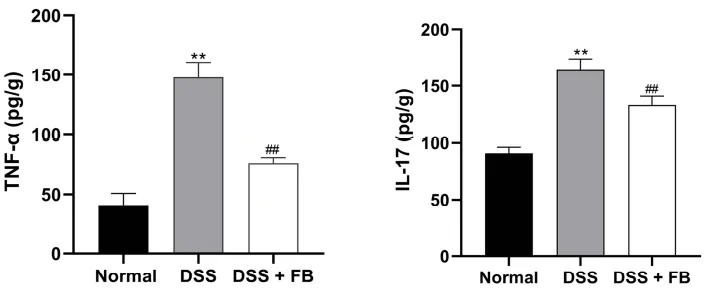
Detecting the levels of inflammatory cytokines in the supernatant by ELISA. All values were expressed as mean ± SD, n = 3. ^∗∗^*p* < 0.01 *vs.* the Normal group; ^##^*p* < 0.01 *vs.* the DSS group.

**Table 1:** Primer sequences used for RT-qPCR.

**Gene**	**Primer Sequences**	**Amplicon Size (Base Pair)**
p38 MAPK	F: 5'-CCGCAAGGTCACTGGAGGAA-3' R:5'-TCGGAGGATCTGGTAGATGAGAAA-3'	134
NF-κB p65	F: 5'-CGCAAAAGGACCTATGAGACC-3' R: 5'-AAGTTGATGGTGCTGAGGGAT-3'	176
HIF-1α	F: 5'-TCTCCAAGCCCTCCAAGTATG-3' R: 5'-GCCTTAGCAGTGGTCGTTTCT-3'	203
ICAM-1	F: 5'-CGTGATGGCAGCCTCTTATGT-3' R: 5'-GGGCTTGTCCCTTGAGTTTTA-3'	101
VCAM-1	F: 5'-TTGTGGAAATGTGCCCGAAAC-3' R: 5'-GTGCCTGGCGGATGGTGTA -3'	104
β-actin	F: 5'-AGCCATGTACGTAGCCATCC-3' R: 5'-CTCTCAGCTGTGGTGGTGAA-3'	227

## Data Availability

The data supporting the findings of this study are provided within the article and the Supplementary Material Tables.

## LIST OF ABBREVIATIONS

CAM-1: Cell Adhesion Molecule 1
GM-CSF: Granulocyte-macrophage Stimulating Factor
GO: Gene Ontology
PMSF: Phenylmethylsulfonyl Fluoride
UC: Ulcerative Colitis

## SUPPLEMENTARY MATERIAL

**Supplemental Table S1.** Basic information of the main active ingredients of FB herb pair.
**Supplemental Table S2.** Information on 34 drug ingredients in network pharmacology.
**Supplemental Table S3.** The topological characteristic of the protein-protein interaction network.
**Supplementary Table S4.** Body weights of mice in each group.
**Supplementary Table S5.** The DAI scores in each group.
**Supplementary Table S6.** The chemical constituents of FB-containing serum identified by UPLC-Q-Orbitrap.
