# Supplementary material for: Fuzi-Baijiangcao Herb Pair Alleviates DSS-induced Ulcerative Colitis in Mice via Inhibiting the p38 MAPK/NF-κB/HIF-1α Signaling Pathway
Source: Comb Chem High Throughput Screen. 2025 Apr 15;29(16):2304–17. doi: 10.2174/0113862073342236250103112654 (PMC13684974; doi:10.2174/0113862073342236250103112654)
Supplement: Supplementary file 1 [file CCHTS-29-16-2304_SD1.pdf]

Supplementary Material

Fuzi-Baijiangcao Herb Pair Alleviates DSS-induced Ulcerative Colitis in Mice *via* Inhibiting the p38 MAPK/NF-κB/HIF-1α Signaling Pathway

Chengxin Sun[1], Yinghao Jiang[1] and Xiaoyan Wang[2,*]

[1]*The First Clinical Medical College, Shandong University of Traditional Chinese Medicine, Jinan, Shandong, China;* [2]*College of Traditional Chinese Medicine, Shandong University of Traditional Chinese Medicine, Jinan, Shandong, China*

Table S1. Basic information of the main active ingredients of FB herb pair.

MolID	Molecule	OB(%)	DL
Fuzi MOL002211	11,14-eicosadienoic acid	39.99	0.2
Fuzi MOL002388	Delphin_qt	57.76	0.28
Fuzi MOL002392	Deltoin	46.69	0.37
Fuzi MOL002393	Demethyldelavaine A	34.52	0.18
Fuzi MOL002394	Demethyldelavaine B	34.52	0.18
Fuzi MOL002395	Deoxyandrographolide	56.3	0.31
Fuzi MOL002397	karakoline	51.73	0.73
Fuzi MOL002398	Karanjin	69.56	0.34
Fuzi MOL002401	Neokadsuranic acid B	43.1	0.85
Fuzi MOL002406	2,7-Dideacetyl-2,7-dibenzoyl-taxayunnanine F	39.43	0.38
Fuzi MOL002410	benzoylnapelline	34.06	0.53
Fuzi MOL002415	6-Demethyldesoline	51.87	0.66
Fuzi MOL002416	deoxyaconitine	30.96	0.24
Fuzi MOL002419	(R)-Norcoclaurine	82.54	0.21

Fuzi<br>MOL002421	ignavine	84.08	0.25
Fuzi<br>MOL002422	isotalatizidine	50.82	0.73
Fuzi<br>MOL002423	jesaconitine	33.41	0.19
Fuzi<br>MOL002433	(3R,8S,9R,10R,13R,14S,17R)-3-hydroxy-4,4,9,13,14-pentamethyl-17-[(E,2R)-6-methyl-7-[(2R,3R,4S,5S,6R)-3,4,5-trihydroxy-6-[[(2R,3R,4S,5S,6R)-3,4,5-trihydroxy-6-(hydroxymethyl)oxan-2-yl]oxymethyl]oxan-2-yl]oxyhept-5-en-2-yl]-1,2,3,7,8,10,12,15,16,17-decahydr	41.52	0.22
Fuzi<br>MOL002434	Carnosifloside I_qt	38.16	0.8
Fuzi<br>MOL000359	sitosterol	36.91	0.75
Fuzi<br>MOL000538	hypaconitine	31.39	0.26
Baijiangcao<br>MOL001676	Vilmorrianine C	33.96	0.22
Baijiangcao<br>MOL001677	asperglaucide	58.02	0.52
Baijiangcao<br>MOL001678	bolusanthol B	39.94	0.41
Baijiangcao<br>MOL001790	Linarin	39.84	0.71
Baijiangcao<br>MOL001689	acacetin	34.97	0.24
Baijiangcao<br>MOL002322	isovitexin	31.29	0.72
Baijiangcao<br>MOL001697	Sinoacutine	63.39	0.53
Baijiangcao<br>MOL000358	beta-sitosterol	36.91	0.75
Baijiangcao<br>MOL000359	sitosterol	36.91	0.75
Baijiangcao<br>MOL000422	kaempferol	41.88	0.24
Baijiangcao<br>MOL000449	Stigmasterol	43.83	0.76
Baijiangcao<br>MOL000006	luteolin	36.16	0.25
Baijiangcao<br>MOL000098	quercetin	46.43	0.28

Table S2. Information on 34 drug ingredients in network pharmacology.

Herbs	MOLID	Molecule name
Baijiangcao	MOL001676	Vilmorrianine C
Baijiangcao	MOL001677	asperglaucide
Baijiangcao	MOL001678	bolusanthol B
Baijiangcao	MOL001790	Linarin
Baijiangcao	MOL001689	acacetin
Baijiangcao	MOL002322	isovitexin
Baijiangcao	MOL001697	Sinoacutine
Baijiangcao	MOL000358	beta-sitosterol
Baijiangcao	MOL000359	sitosterol
Baijiangcao	MOL000422	kaempferol
Baijiangcao	MOL000449	Stigmasterol
Baijiangcao	MOL000006	luteolin
Baijiangcao	MOL000098	quercetin
Fuzi	MOL002211	MOL002211
Fuzi	MOL002388	MOL002388
Fuzi	MOL002392	MOL002392
Fuzi	MOL002393	MOL002393
Fuzi	MOL002394	MOL002394
Fuzi	MOL002395	MOL002395
Fuzi	MOL002397	MOL002397
Fuzi	MOL002398	MOL002398
Fuzi	MOL002401	MOL002401
Fuzi	MOL002406	MOL002406
Fuzi	MOL002410	MOL002410
Fuzi	MOL002415	MOL002415
Fuzi	MOL002416	MOL002416
Fuzi	MOL002419	MOL002419
Fuzi	MOL002421	MOL002421
Fuzi	MOL002422	MOL002422
Fuzi	MOL002423	MOL002423
Fuzi	MOL002433	MOL002433
Fuzi	MOL002434	MOL002434
Fuzi	MOL000359	MOL000359
Fuzi	MOL000538	MOL000538

Table S3. The topological characteristic of the protein-protein interaction network.

Protein	Degree	Neighborhood Connectivity	Topological Coefficient
IL1B	51	18.35294118	0.210953347
JUN	47	18.72340426	0.214966985
MMP9	44	20.15909091	0.231713689
HIF1A	41	19.29268293	0.231854246
VCAM1	36	21.97222222	0.26795393
ICAM1	34	23.05882353	0.288235294
PPARG	33	20.66666667	0.246031746
ERBB2	31	20.41935484	0.2520908
CAV1	29	21.75862069	0.259031199
SERPINE1	25	24.8	0.31
STAT1	25	24.24	0.306835443
CXCL10	24	24.375	0.325
JAK2	24	23.875	0.30952381
TGFB1	23	28.04347826	0.354980737
HMOX1	23	24.86956522	0.331594203
MMP1	22	25.68181818	0.342424242
NOS2	22	24	0.306526807
SPP1	21	27.42857143	0.356215213
PLAU	21	25.52380952	0.349641226
MMP3	20	25.85	0.354109589
MMP7	20	25.7	0.338157895
CXCL2	19	24.47368421	0.359907121
CCR2	18	25.44444444	0.334795322
CSF1R	18	23.66666667	0.314074074
COL1A1	18	25.66666667	0.340740741
KIT	18	26.33333333	0.355855856
CCR5	17	25.11764706	0.358823529
PDGFRB	16	27.5625	0.353365385
CTSB	15	20.26666667	0.301492537
CTSD	14	20.78571429	0.304621849
DPP4	14	25.07142857	0.352112676
CDK1	14	13.85714286	0.202731092
CTSK	14	22.71428571	0.319919517
CTSL	13	19.53846154	0.299408284
IRF1	13	29.69230769	0.395897436
CXCL11	13	25.38461538	0.430247718

CCR1	13	25.23076923	0.406947891
FYN	13	22.46153846	0.298461538
HSPA5	13	25.23076923	0.327672328
GJA1	13	27.53846154	0.356643357
PLAT	12	25.66666667	0.442528736
F3	12	25.83333333	0.385572139
RXRA	12	12	0.176616915
CCNA2	12	18.66666667	0.262910798
NCOA2	12	17.41666667	0.246428571
CYP3A4	12	9	0.16509434
PARP1	11	22.72727273	0.299043062
CCNB1	11	20.18181818	0.28425096
AHR	11	27.09090909	0.35645933
NR1I2	11	18.72727273	0.256537983
THBD	10	28.2	0.486206897
PIK3CD	9	18.88888889	0.325670498
HSPB1	9	29.22222222	0.38962963
ALOX5	9	27.88888889	0.435763889
ERBB3	8	28.375	0.417279412
BIRC5	8	15.25	0.287735849
CPT1A	7	17.14285714	0.281030445
ABCB1	7	27	0.391304348
NR1H3	6	27	0.397058824
INSR	6	22.16666667	0.388888889
TK1	6	11	0.333333333
PTGS1	6	22	0.35483871
HSD11B1	5	12.8	0.290909091
PTAFR	5	25.6	0.465454545
NR3C2	5	14.6	0.306382979
PLA2G2A	5	18	0.285714286
DUOX2	4	35	0.564516129
SLPI	4	30.75	0.521186441
CPT2	4	16	0.4
H1F0	4	12	0.6
CA2	4	26.5	0.452586207
CDC25B	4	11.25	0.661764706
ODC1	3	23.33333333	0.442307692
PDE5A	3	12	0.35483871

PNP	3	8.666666667	0.348484848
CTSH	3	15	0.47311828
CHKA	3	19	0.513513514
PIM1	3	17	0.387596899
GABRA2	3	6.333333333	0.375
MAOA	3	6	0.377777778
ADH1C	3	2.666666667	0.466666667
PDE3A	2	2	0.5
PIM2	2	13.5	0.520833333
HSD11B2	2	5	0.625
EPHX2	2	12.5	0.522727273
DHCR7	2	12.5	0.5
CES2	2	11.5	0.638888889
CA12	2	21	0.5
AKR1B10	2	2	0.5
PDE4B	1	2	0
PSMB5	1	3	0
MAP3K12	1	47	0
EGLN1	1	41	0
SLC5A1	1	14	0
PCOLCE	1	18	0
CA4	1	2	0
CA1	1	4	0
BACE2	1	15	0
GLA	1	2	0

Table S4. Body weights of mice in each group (n=6).

Days	Normal	DSS	DSS+FB
1	19.33±0.89	19.20±0.61	19.75±3.99
2	19.65±085	19.23±0.64	19.77±4.22
3	20.05±0.75	19.21±0.63	19.65±3.99
4	20.48±0.98	19.10±0.65	19.63±0.37
5	20.67±0.87	18.97±0.66	19.67±0.34
6	20.91±0.95	18.92±0.66	19.62±0.31
7	21.17±1.04**	18.40±0.59	19.05±0.35
8	21.27±1.14**	17.62±0.87	18.62±0.52
9	21.48±1.31**	16.07±0.56	17.08±0.99

10	21.33±1.30**	14.97±0.48	15.78±0.55
11	21.68±1.21**	14.17±0.56	15.15±0.95
12	22.37±1.01**	13.73±0.34	14.63±0.83 #
13	22.40±1.09**	13.55±0.21	15.18±1.15 ##
14	22.58±1.29**	13.35±0.11	15.78±1.39 ##
15	22.40±1.09**	13.45±0.45	16.48±1.12 ##

** $p < 0.01$, Normal *vs*. DSS group; # $p < 0.05$, ## $p < 0.01$, DSS+FB *vs*. DSS group.

Table S5. The DAI scores in each group (n=6).

Days	Normal	DSS	DSS+FB
1	0	0	0
2	0	0.11±0.17	0.11±0.17
3	0	0.22±0.18	0.22±0.17
4	0	0.32±0.30	0.33±0.30
5	0	0.67±0.30	0.44±0.18
6	0	0.78±0.17	0.56±0.18
7	0	1.22±0.17	1.22±0.35
8	0	1.44±0.18	1.44±0.18
9	0	1.78±0.17	1.89±0.17
10	0	1.44±0.18	1.56±0.18
11	0	1.33±0.30	1.33±0.10
12	0	1.22±0.17	1.11±0.17
13	0	1.00±0.21	0.78±0.17#
14	0	0.89±0.17	0.67±0.10#
15	0	0.56±0.18	0.22±0.17##

# $p < 0.05$, ## $p < 0.01$, DSS+FB *vs*. DSS group.

Table S6. The chemical constituents of FB-containing serum identified by UPLC-Q-Orbitrap.

Number	Name	Formula	Molecular Weight	RT (min)
1	DL-Homoserine	C4 H9 N O3	119.05851	1.31
2	D-(+)-Malic acid	C4 H6 O5	134.02026	1.808
3	Epinephrine	C9 H13 N O3	183.08882	10.746
4	Deoxycholic acid	C24 H40 O4	392.29271	19.963
5	Pinolenic acid	C18 H30 O2	278.2245	21.486
6	Monoolein	C21 H40 O4	356.29219	22.166
7	Stearic Acid	C18 H36 O2	284.27167	23.93
